# IC_50_: an unsuitable measure for large-sized prostate cancer spheroids in drug sensitivity evaluation

**DOI:** 10.17305/bjbms.2022.7279

**Published:** 2022-06-09

**Authors:** Yipeng Xu, Gabriela Pachnikova, He Wang, Yaoyao Wu, Dorothea Przybilla, Reinhold Schäfer, Zihao Chen, Shaoxing Zhu, Ulrich Keilholz

**Affiliations:** 1 Department of Urology, The Cancer Hospital of the University of Chinese Academy of Sciences (Zhejiang Cancer Hospital), Hangzhou, China; 2 The Key Laboratory of Zhejiang Province for Aptamers and Theranostics, Hangzhou, China; 3 Institute of Basic Medicine and Cancer, Chinese Academy of Sciences, Hangzhou, China; 4 Comprehensive Cancer Center, Charité – Universitätsmedizin Berlin, Corporate Member of Freie Universität Berlin, Humboldt-Universität zu Berlin and Berlin Institute of Health, Berlin, Germany; 5 The Second Clinical Medical College, Zhejiang Chinese Medical University, Zhejiang, China; 6 Department of Urology, Southern Medical University, Guangzhou, China; 7 German Cancer Consortium (DKTK), Heidelberg, Germany

**Keywords:** IC_50_, docetaxel, spheroids, drug testing, preclinical models, prostate cancer, 2D clinical models, 3D clinical models, cell culture

## Abstract

Preclinical models of tumors have the potential to become valuable tools for commercial drug research and development, and 3D culture systems are gaining traction in this area, particularly in prostate cancer (PCa) research. However, nearly all 3D drug design and screening assessments are based on 2D experiments, suggesting limitations of 3D drug testing. To simulate the natural response of human cells to the drug, we detected the half-maximal inhibitory concentration (IC_50_ ) changes of 2D/3D LNCaP cells in the drug docetaxel, as well as the sensitivity of different morphologies of 2D/3D LNCaP to docetaxel treatment. In contrast to 2D LNCaP cells, the evaluation of LNCaP spheroids’ susceptibility to treatment was more complicated; the fitness of IC_50_ curves of 2D and 3D tumor cell preclinical models differs significantly. IC_50_ curves were unsuitable for large-sized LNCaP spheroids. More evaluation indexes (such as max inhibition) and experiments (such as spheroids formation) should be explored and performed to evaluate the susceptibility systematically.

## INTRODUCTION

Despite decades of tremendous progress in commercial drug research and development, the discovery of novel effective drugs has decreased[1]. It has been reported that novel oncology drugs have lower success rates than drugs for other diseases throughout late clinical development stages [2]. The main reason for this low success rate was suggested to be a lack of efficacy[3]. Clinical trials are based on preclinical model evidence of effectiveness. Therefore, a preclinical model that more accurately mimics human tumors would be a useful tool for translational research of anti-cancer drugs.

Today, most preclinical prostate cancer (PCa) research is still undertaken in 2D-cultured PCa cell lines, the most common of which being PC3, DU145 and LNCaP [4]. The advantages of 2D-cultured PCa cell lines are ease of use, high reproducibility, and cost-effectiveness[5]. However, cell lines may accumulate several additional mutations due to a long-term culture. Therefore, data generalization for clinical practice might be challenging due to a lack of essential characteristics. For example, two of the PCa cell lines widely used in research, DU145 and PC3, do not express androgen receptor (AR) and prostate-specific antigen (PSA)[4, 5], which may play an important role in drug response.

On the other hand, compared to cell lines, patient-derived xenografts (PDXs) reflect the cellular heterogeneity and molecular divergence normally present in a tumor environment more appropriately [6, 7]. PDXs of PCa were shown to be beneficial in drug screenings for efficacy and toxicity[8]. However, given the low engraftment rate (15-20%), high costs and long experimental periods (usually several months), the possible standardized use of PDXs of PCa is challenging[9].

The murine tumor microenvironment only partially reflects the human tumor microenvironment. Original tumor samples obtained through surgical resections are the primary source of PDXs, and obtaining them repeatedly is extremely complicated. As the passages of the PDX model progress, the original tumor microenvironment is gradually replaced by a mouse-derived matrix, reducing the PDX passage numbers in further applications[10].

Given the limitations of 2D-cultured cell lines and PDX models, 3D cell culture systems such as organoids and spheroids are receiving more attention in PCa research. Both organoids and spheroids are regarded as the intermediate models between *in vitro* 2D-cultured cell lines and PDXs. Spheroids are often made from cancer cell lines or tumor biopsies, whereas organoids are derived from primary tumor tissues[11]. Organoids offer a higher complexity and are more *in vivo*-like than spheroids. Unfortunately, the efficiency for PCa organoids establishment is only 15-20%[12]. Compared to PCa organoids, the efficiency of establishing PCa spheroids based on PCa cell lines is much higher, allowing them to be widely used models in novel anti-cancer drug therapies[13], studies of treatment-induced drug resistance[14], and drug screening[15].

Despite the advantages of spheroids over 2D-cultured cells and animal models, their use as preclinical models for drug testing is still limited. There are multiple challenges to be addressed for spheroids experimental protocols to be widely adopted and employed in fundamental research and drug screening[16]. Colorimetric, luminescence, or fluorescence assays, which were initially developed for 2D-monolayer cultured cells, are also used to analyze drug testing experiments based on spheroids. The evaluation indexes of drug effectiveness for spheroids are based on the IC_50_ values generated from 2D monolayer cultured cells. Spheroids have been shown to be substantially more drug-resistant than 2D cultivated cells in several studies [17]. A new evaluation system or combined evaluation indexes should be investigated for spheroids drug testing experiments.

We harvested spheroids from the LNCaP cell line and then evaluated the docetaxel treatment for 2D cultured cells and different-sized 3D preclinical models of prostate cancer to evaluate if the IC_50_ values are suitable for spheroids drug testing experiments.

## MATERIALS AND METHODS

### Cell culture

We obtained the LNCaP cell line from the Urology Department of Charité Campus Mitte. All the 2D and 3D cultured cells were cultured at a humidified incubator with 5% CO_2_ at 37^o^C (Thermo Scientific; Massachusetts, USA). The 2D and 3D cultured LNCaP cells were maintained in complete growth RPMI medium (Gibco; Texas, USA) supplemented with 10% FBS (Gibco; Texas, USA) and 1% Penicillin and Streptomycin (stock 10000 μg/ml each) (Life Technologies; New York, USA) according to the ATCC website (https://www.atcc.org/).

### 3D cells/spheroids workflow

**FIGURE 1:**
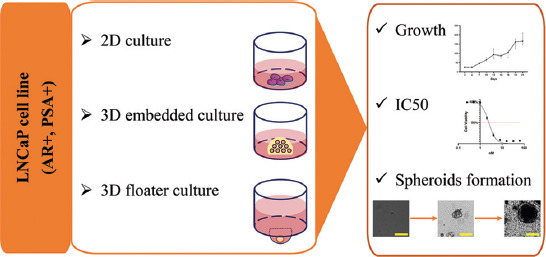
The 2D/3D preclinical models of prostate cancer workflow. AR: androgen receptor; PSA: prostate-specific antigen; IC_50_: half-maximal inhibitory concentration.

In this research, the LNCaP cells were set up as 2D or 3D preclinical models. Two kinds of 3D culture protocols were used in this study: 3D embedded culture and 3D floater culture (Figure 1). The growth of the 3D cultured cells/spheroids was monitored via TCS SPE confocal system microscope images of LNCaP cells/spheroids (Leica; Germany), and the susceptibilities of LNCaP cells/spheroids were evaluated by spheroids formation and IC_50_ curves.

#### 3D embedded cells/spheroids culture

The 3D-cultured LNCaP cells/spheroids were initiated as a 2D-cultured monolayer. From the 3^rd^ passages on after cell thawing, a certain number of 2D-cultured LNCaP cells were resuspended in the Matrigel Matrix (Corning; New York, USA) and plated into the center of each well of TC-treated 96 well microplates (Corning; New York, USA). The medium was exchanged every three days for models seeded in 24 well plates, and every second day for the 96 well plates.

#### 3D floater culture

Unlike the 3D-embedded cells/spheroids culture, the cells/spheroids in this protocol were floaters at the U-bottom of each well instead of embedded in Matrigel Matrix. The low attachment U-bottom 96 well spheroids microplates (Corning; New York, USA) were used in the cell culture experiments, and drug tests work. From the 3^rd^ passages on after cell thawing, a certain number of 2D-cultured LNCaP cells were resuspended in the prewarmed RPMI medium supplemented with 10% FBS and 1% P/S. 200 μl of this cell suspension were added per one well into the 96 well spheroids microplates. The medium was exchanged every second day by exchanging only half of the medium volume for a fresh one and keeping half of the volume of the medium from the previous cultivation time.

### 2D drug testing experiments

100 μl of the 2D-cultured LNCaP cells were collected and seeded into one well using the 96 well TC-treated microplates (100 μl/well). These were placed in the CO_2_ incubator for 48 hours for the cells to grow. Treatment was done after this time period by adding 100 μl of fresh RPMI medium supplemented with 10% FBS and 1% P/S with the 2× docetaxel (AbCam; Cambridge, UK) as a final concentration (0.125 nM, 0.25 nM, 0.5 nM, 1 nM, 2 nM, 4 nM, 8 nM, 16 nM, and 32 nM). The negative control contained no drug. On the 6^th^ day of the cell cultivation with or without drug treatment, the CellTiter Glo assay (Promega; Wisconsin, USA) was performed according to the producer´s instructions and the cell viability after treatment was read by VICTOR Nivo Multimode Microplate Reader (PerkinElmer, Massachusetts, USA).

### 3D drug testing experiments based on embedded cultured LNCaP cells/spheroids

The 2D-cultured LNCaP cells were seeded into the 96 well TC-treated microplates. Prewarmed complete growth medium (as mentioned above) was gently added into each well (200 μl/well). Plates were placed in the CO_2_ incubator for 2, 4, 7, 14, and 21 days to obtain different-sized spheroids. The growth medium was exchanged every second day (100 μl/well). Treatment was done as follows: double the concentration of docetaxel was added in 100 μl of fresh medium. This was added as a replacement for 100 μl of old medium per well. In addition, 100 μl of the old medium was kept in each well so that 1× concentration (0.25 nM, 0.5 nM, 1 nM, 2 nM, 4 nM, 8 nM, 16 nM, 32 nM, 64 nM, 128 nM, 256 nM, 512 nM, and 1024 nM) of drug was achieved. The half-old and half-new medium approach was used to help cells grow at least partially under a stable condition. No drug presence in the medium was used as a negative control. On the 6^th^ day of cell culture with or without drug treatment, the CellTiter Glo assay was performed according to the producer´s instructions and the cell viability after treatment was read by VICTOR Nivo Multimode Microplate Reader.

### 3D drug testing experiments based on different-sized floating LNCaP spheroids

The 2D-cultured LNCaP cells were collected and seeded into a spheroids microplate (300 or 3000 cells per well) in 200 μl of growth medium (as mentioned earlier). Cells were cultured like this for 48 hours under sterile conditions in a 37^o^C incubator with 5% CO_2_. After this, treatment with docetaxel was administered as mentioned in the section 2.4 above. On the 6^th^ day of cell culture with or without drug treatment, the CellTiter Glo assay was performed according to the producer´s instructions and the cell viability, after treatment was read by VICTOR Nivo Multimode Microplate Reader.

### Statistical analysis

The normality of LNCaP cells/spheroids size, including frequency distribution and Gaussian distribution (D’Agostino-Pearson omnibus normality test), the spheroids parameters (d_max_, spheroids volume, and lg volume, all as violin plots), as well as IC_50_ values were analyzed by GraphPad Prism 8 software (GraphPad Software; California, USA).

## RESULTS

### Formation inconsistency of LNCaP spheroids in Matrigel Matrix

**FIGURE 2:**
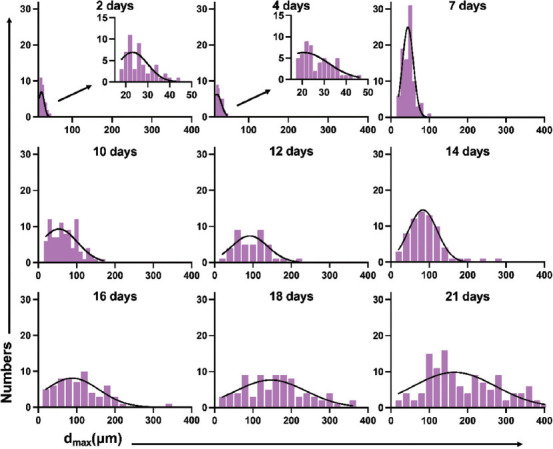
Histogram of d_max_ of LNCaP spheroids from day 2 to day 21. d_max_: Major diameter.

**FIGURE 3:**
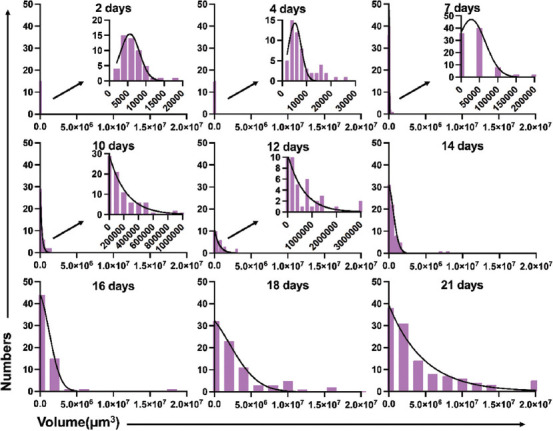
Histogram of the volume of LNCaP spheroids from day 2 to day 21.

**FIGURE 4:**
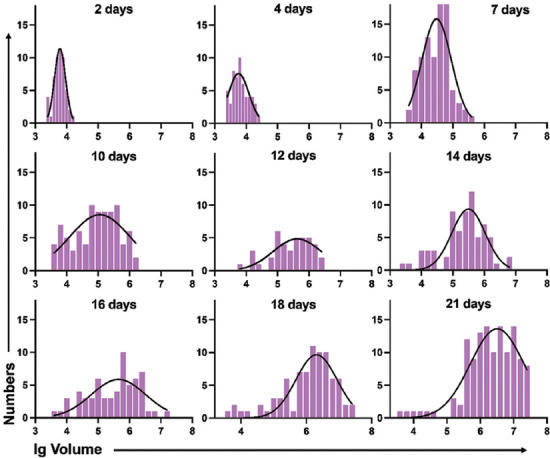
Histogram of lg volume of LNCaP spheroids from day 2 to day 21. lg volume: log_10_ volume

Morphological changes from single cells to spheroids could be observed under the microscope over a certain period of time. To clarify the consistency of LNCaP spheroids sizes, we collected three parameters (major diameter/d_max_, spheroids volume, and lg volume) at different time points (Figure 2-4).

#### Formation inconsistency of LNCaP spheroids based on major diameter (d_max_)

We chose to use a histogram (Figure 2) to show the distribution of spheroids d_max_. Frequency distribution and D’Agostino-Pearson omnibus normality tests were performed to determine whether the d_max_ fit a normal distribution. We found that all the d_max_ data at different time points passed the normality test (P>0.05).

#### Formation inconsistency of LNCaP spheroids based on spheroids volume

Another parameter that represents the size of LNCaP spheroids is volume. According to the literature[18], the LNCaP spheroid volume can be calculated as:


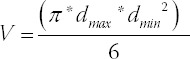


We also used the histogram (Figure 3) to show the distribution of spheroids’ volume. Frequency distribution and D’Agostino-Pearson omnibus normality tests showed that the data on days 10, 16, 18, and 21 did not pass the normality test (P<0.05) (Table 1).

**TABLE 1:**
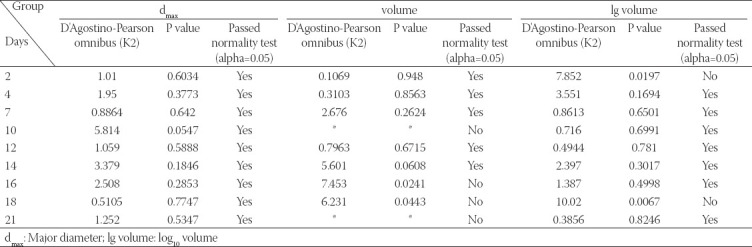
The results of the normality tests for LNCaP cells/spheroids at different times

#### Formation inconsistency of LNCaP spheroids based on spheroids’ lg volume

The results above demonstrate that the LNCaP spheroids volumes did not pass the normality test at some time points, especially when the spheroids were large. One explanation might be the long span of spheroids volume by time. Therefore, we performed frequency distribution and D’Agostino-Pearson omnibus normality tests to evaluate whether the lg volume (log_10_ volume) values fit the normal distribution (Figure 4). Almost all of the lg volume of the LNCaP spheroids passed the normality test at different time points, except day 2 and day 18.

#### Variations in the size of the LNCaP cells/spheroids distribution

**FIGURE 5:**
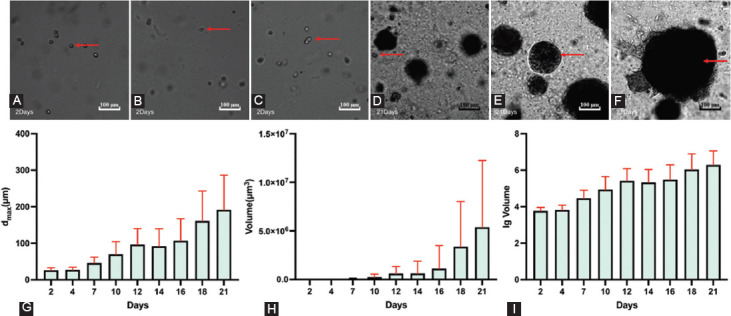
. Images of LNCaP cells and spheroids on day 2 and day 21 and growth kinetics of 3D-embedded LNCaP cells/spheroids. (A) Image of the smallest LNCaP cell on day 2 (red arrow). (B) Image of the median LNCaP cell on day 2 (red arrow). (C) Image of the biggest LNCaP cell on day 2 (red arrow). (D) Image of the smallest LNCaP cell and spheroid on the day 21 (red arrow, still single cell). (E) Image of the median LNCaP cell and spheroid on day 21 (red arrow). (F) Image of the biggest LNCaP cell and spheroid on the day 21 (red arrow). (G) The violin plots of 3D-embedded LNCaP cells/spheroids based on d_max_. (H) The violin plots of 3D-embedded LNCaP cells/spheroids based on cells/spheroids volume. (I) The violin plots of 3D-embedded LNCaP cells/spheroids based on cells/spheroids’ lg volume. d_max_: Major diameter; lg volume: log_10_ volume

According to the results above, we found that the peak of each parameter’s distribution moves from left to right (small size to large size) over time. Initially, almost all of the cells derived from single cells, and majority of them had developed into spheroids at day 21. The volume difference between the biggest and smallest LNCaP spheroids was 7.241-fold (2377 vs. 17214 μm^3^) on the second day and 8433-fold (3730 vs. 31465801 μm^3^) on the twenty-first day. Even though the percentage of single cells was relatively low, single LNCaP cells were still detectable on day 21 (Figure 5D). For the three parameters we used to describe the spheroids’ size, the mean value of d_max_ for LNCaP spheroids at day 21 became 6.587-fold higher than that of single LNCaP cells (168.5 vs. 25.58 μm). The difference between the lg volume of spheroids was only a 1.681-fold increased (6.360 vs. 3.784). The frequency distributions and D’Agostino-Pearson omnibus normality tests described above showed that most d_max_ and lg volume values were normally distributed, while 4/9 data on the spheroids volume did not pass the normality tests. Based on the results above, we chose each parameter’s median to describe the size of the LNCaP cells/spheroids in the following evaluation of LNCaP cells/spheroids growth kinetics.

### Growth kinetics of 3D-embedded LNCaP cells/spheroids

In contrast to the proliferation of 2D-cultured cells, the growth of LNCaP cells/spheroids in Matrigel Matrix was evaluated to increase spheroids’ size (Figure 5A-F). To show the continuous changes of the LNCaP cells/spheroids and to describe the size of the LNCaP spheroids, we used three parameters (major diameter/d_max_, spheroids volume, and lg volume). The frequency distribution was represented by the violin plots. Since not all data were normally distributed, the median was utilized to describe the size of the LNCaP spheroids instead of the mean.

As shown in Figure 5G (the violin plots of d_max_), and the cells/spheroids images below, the LNCaP spheroids were initiated from single LNCaP cells, and were still in a single state at the day 2 and 4 (Figure 5A-C). The formation of LNCaP spheroids could have been observed from the seventh day on. The images represent the median-sized spheroids at various time points. The violin plots based on spheroids volume (Figure 5H) could not initially display the cells/spheroids since the volume span was much greater than the d_max_, resulting in volume too close to the X-axis. The lg volume of the LNCaP cells/spheroids were also evaluated to show the growth kinetics, which seems to be a useful parameter to display the LNCaP cells/spheroids and evaluate growth kinetics (Figure 5I).

### Susceptibility of LNCaP cells/spheroids to docetaxel treatment

#### Susceptibility of 2D/3D LNCaP cells to docetaxel treatment

**FIGURE 6:**
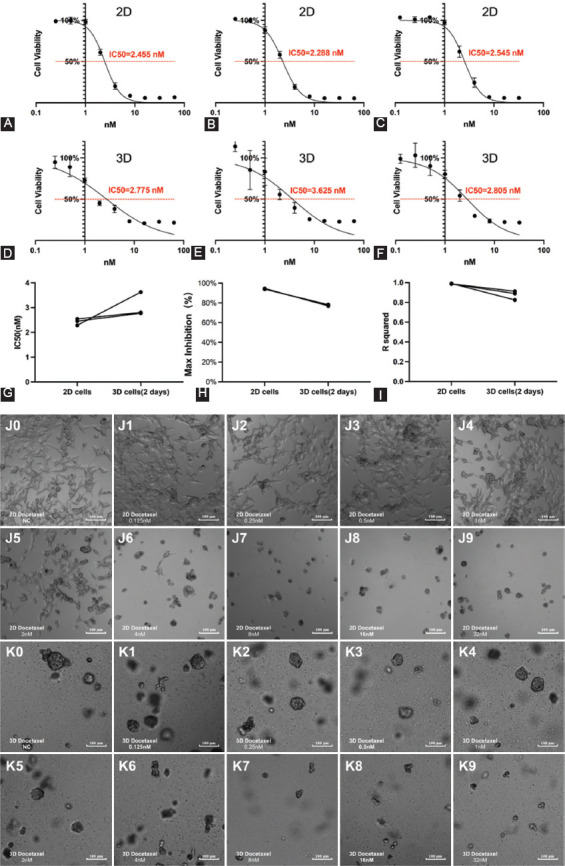
Susceptibility of 2D/3D LNCaP cells exposed to docetaxel treatment. (A-C) The drug testing experiments of 2D cultured LNCaP cells exposed to varying docetaxel concentrations: The IC_50_ values were 2.455 nM, 2.288 nM, and 2.545 nM. (D-F) The drug testing experiments of 3D-cultured LNCaP cells exposed to varying docetaxel concentrations: The IC_50_ values were 2.775 nM, 3.625 nM, and 2.805 nM. (G) The results of the permutation test of IC_50_. (H) Maximal inhibition values. (I) R2 values. (J0-J9) Images of the drug testing experiments based on 2D-cultured LNCaP cells exposed to varying docetaxel concentrations: (J0) Image of negative control without docetaxel; (J1-J9) Image of the LNCaP cells exposed to varying docetaxel concentrations for 5 days (0.125 nM, 0.25 nM, 0.5 nM, 1 nM, 2 nM, 4 nM, 8 nM, 16 nM, and 32 nM). (K0-K9) Images of the drug testing experiments based on 3D-cultured LNCaP cells exposed to varying docetaxel concentrations: K0: Image of negative control without docetaxel; K1-K9: Image of the LNCaP cells exposed to varying docetaxel concentrations for 5 days (0.125 nM, 0.25 nM, 0.5 nM, 1 nM, 2 nM, 4 nM, 8 nM, 16 nM, and 32 nM). IC_50_: half-maximal inhibitory concentration; d_max_: Major diameter; lg volume: log_10_ volume.

**FIGURE 7:**
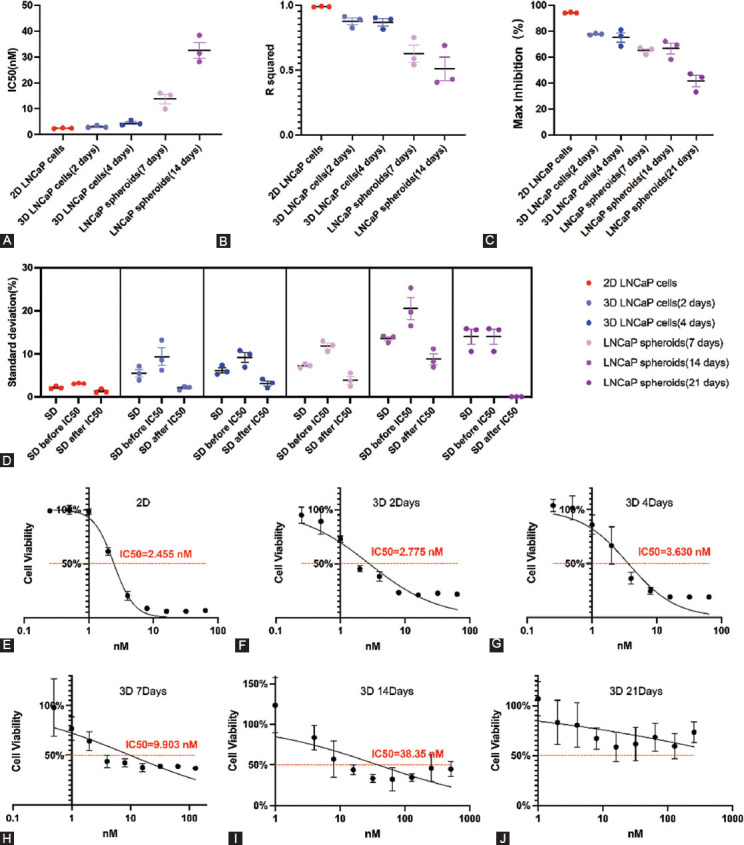
The parameters of the drug testing experiments based on 2D LNCaP cells and 3D-embedded cultured LNCaP cells/spheroids exposed to docetaxel and the IC50 curves with the median R2 values. (A) The IC_50_ values of 2D LNCaP cells and 3D-embedded cultured LNCaP cells/spheroids exposed to docetaxel. (B) The R2 values of 2D LNCaP cells and 3D-embedded cultured LNCaP cells/spheroids exposed to docetaxel. (C) The maximal inhibition of 2D LNCaP cells and 3D-embedded cultured LNCaP cells/spheroids exposed to docetaxel. (D) The SD values of 2D LNCaP cells and 3D-embedded cultured LNCaP cells/spheroids exposed to docetaxel. (E-J) The IC_50_ curves with the median R2 values: E: The IC_50_ curve of 2D LNCaP cells exposed to docetaxel; (F) The IC_50_ curve of 3D-embedded cultured LNCaP cells(cultured for 2 days before plating); (G) The IC_50_ curve of 3D-embedded cultured LNCaP cells(cultured for 4 days before plating); (H) The IC_50_ curve of 3D-embedded cultured LNCaP spheroids(cultured for 7 days before plating); (I) The IC_50_ curve of 3D-embedded cultured LNCaP spheroids(cultured for 14 days before plating); (J) The IC_50_ curve of 3D-embedded cultured LNCaP spheroids (cultured for 21 days before plating). IC_50_: half-maximal inhibitory concentration.

The IC_50_ values and R^2^ were analyzed using GraphPad Prism 8 software. The maximum inhibition concentrations were averaged according to the last three cell viabilities at the second plateau phase in Excel 2016 (Microsoft; Washington, USA). All IC_50_ values of 3D-cultured LNCaP cells were slightly higher than those of corresponding 2D-cultured LNCaP cells (Figure 6G). Images of the cells showed that the proliferation of 2D-cultured cells was significantly inhibited by docetaxel at high concentrations (Figure 6 J0-J9). In contrast, the inhibition of 3D-cultured cells was reflected both in cell numbers and spheroids sizes (Figure 6 K0-K9). Almost no 2D-cultured cells were living after 5 days of exposure to 32 nM docetaxel (Figure 6 J9), while some living 3D-cultured cells could still be observed (Figure 6 K9). In the IC_50_ curves, the second plateau phase of the 3D-cultured cells (Figure 6D-6F) was much higher than the 2D-cultured cells (Figure 6A-6C), and the IC_50_ curves of the 3D-cultured cells were much flatter than those of the 2D-cultured cells (Figure 7). We further analyzed the maximum inhibition (the mean values of the cell inhibitions at the highest three concentrations at the second plateau phase) and R^2^ (quantifying the goodness of fit) for each IC_50_ curve. The max inhibition values of 3D drug testing experiments were lower than corresponding 2D drug testing experiments (Figure 6H), and the R^2^ values of 3D drug testing experiments were lower as well (Figure 6I).

#### The parameters of 2D and 3D embedded cultured cells as well as spheroids in drug testing experiments

The IC_50_ values and R^2^ values based on 2D LNCaP cells and 3D-embedded cultured LNCaP cells were roughly the same. However, the 3D-embedded cultured LNCaP cells’ maximum inhibition values were much lower than the 2D LNCaP cells. Additionally, we found that the IC_50_ values and SD values became higher by the cells/spheroids size (Figure 7A, D), while the R^2^ values and maximum inhibition became lower (Figure 7B, C). The SD values after IC_50_ is lower than those before IC_50_ (Figure 7D). Combined with the drug testing images, we concluded that this was because most of the small-sized LNCaP spheroids died, resulting in the reduction of data volatility in different wells. The IC_50_ curves also became flatter by the cells/spheroids size (Figure 7E-J), and the lower R^2^ values of the larger-sized spheroids also indicated poor goodness of fit.

#### Susceptibility of floating LNCaP spheroids of variable-sized exposed to docetaxel treatment

**FIGURE 8:**
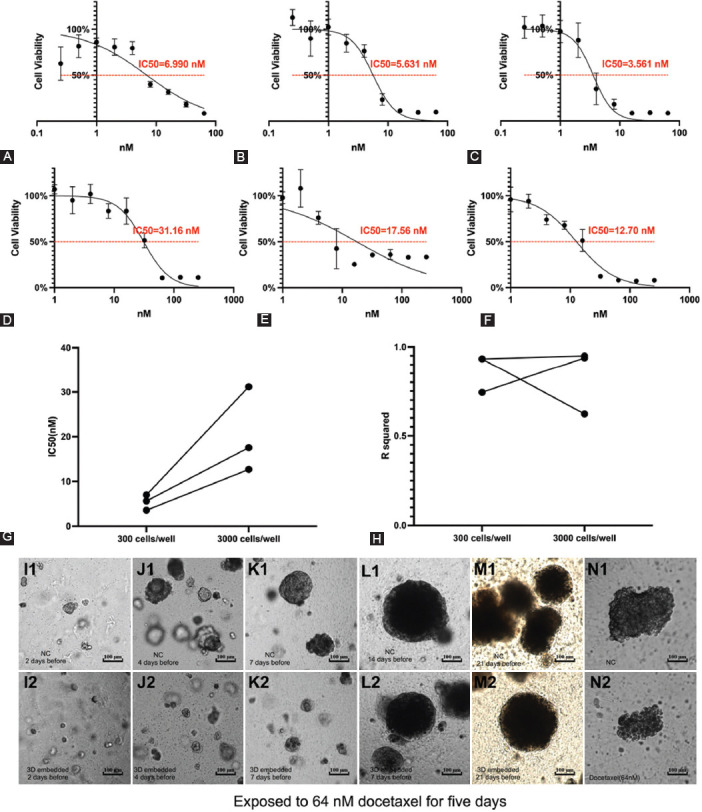
Susceptibility of floating LNCaP spheroids of variable sizes exposed to docetaxel treatment and images of LNCaP cells and spheroids exposed to 64 nM docetaxel treatment. (A-C) The drug testing experiments of smaller LNCaP spheroids (300 cells/well while plating) exposed to varying docetaxel concentrations: The IC_50_ values were 6.990 nM, 5.631 nM, and 3.561 nM. (D-F) The drug testing experiments of bigger LNCaP spheroids (3000 cells/well while plating) exposed to varying docetaxel concentrations: The IC_50_ values were 31.16 nM, 17.56 nM, and 12.70 nM. (G) The IC_50_ values. (H) The R_2_ values. (I1) Image of 3D-embedded cultured LNCaP cells (cultured for 2 days before docetaxel addition) in negative control wells; (I2) Image of 3D-embedded cultured LNCaP cells (cultured for 2 days before docetaxel addition) exposed to 64 nM docetaxel for 5 days. (J1) Image of 3D-embedded cultured LNCaP cells (cultured for 4 days before docetaxel addition) in negative control wells; (J2) Image of 3D-embedded cultured LNCaP cells (cultured for 4 days before docetaxel addition) exposed to 64 nM docetaxel for 5 days. (K1) Image of 3D-embedded cultured LNCaP spheroids (cultured for 7 days before docetaxel addition) in negative control wells; (K2) Image of 3D-embedded cultured LNCaP spheroids (cultured for 7 days before docetaxel addition) exposed to 64 nM docetaxel for 5 days. (L1) Image of 3D-embedded cultured LNCaP spheroids (cultured for 14 days before docetaxel addition) in negative control wells; (L2) Image of 3D-embedded cultured LNCaP spheroids (cultured for 14 days before docetaxel addition) exposed to 64 nM docetaxel for 5 days. (M1) Image of 3D-embedded cultured LNCaP spheroids (cultured for 21 days before docetaxel addition) in negative control wells; (M2) Image of 3D-embedded cultured LNCaP spheroids (cultured for 21 days before docetaxel addition) exposed to 64 nM docetaxel for 5 days. (N1) Image of floating LNCaP spheroids in negative control wells; (N2) Image of floating LNCaP spheroids exposed to 64 nM docetaxel for 5 days. IC_50_: half-maximal inhibitory concentration.

Two types of LNCaP spheroids were acquired based on 300 and 3000 LNCaP cells in the same spheroids microplates and all the parameters are shown in Figure 8A-8H. The experiments were performed in triplicate and showed that the IC_50_ values of bigger LNCaP spheroids were higher than those of smaller spheroids. The R^2^ values of bigger LNCaP spheroids were 0.9378, 0.6223, and 0.949, while the R^2^ values for smaller LNCaP spheroids were 0.7433, 0.9319, and 0.9327. The maximum inhibition values of the bigger and smaller LNCaP spheroids in the first and third experiments showed similar results (Figure 8A vs. D, C vs. F), while the second experiment showed a higher maximum inhibition in the bigger spheroids group (Figure 8 B vs. E). The images demonstrated that the growth of the LNCaP spheroids in size was inhibited by docetaxel treatment. We observed that some LNCaP spheroids exposed to higher docetaxel concentrations (Supplemental Figure 7) appeared loose and flat as well. In these cases, the size of the spheroids measured by the microscope could not precisely demonstrate the cell viability.

#### Imaging of the embedded cultured spheroids and floating spheroids

Two kinds of LNCaP spheroids were used in the drug testing experiments mentioned above: embedded cultured LNCaP spheroids (Figure 8I1-2, 8J1-2, 8K1-2, 8L1-2, 8M1-2) and floating spheroids (Figure 8N1-2). We also compared the images of embedded cultured spheroids and the floating spheroids exposed to 64 nM docetaxel. The floating spheroids became loose and flat when exposed to 64 nM docetaxel for five days (Figure 8N2). On the other hand, the embedded LNCaP spheroids of a similar size exposed to 64 nM docetaxel (Figure 8M2) seemed roughly the same as the spheroids in negative control wells (Figure 8M1). Those loose and flat spheroids were also observed in the small-sized spheroids group (Figure 8J2, K2). They seemed to be more drug-sensitive than large-sized spheroids in the drug testing results.

## DISCUSSION

Establishing preclinical cancer models that can accurately mimic the *in vivo* cancer is crucial for novel anti-cancer drug research and development[4]. Compared to 2D-cultured cell lines, 3D organoids/spheroids culture systems and the PDX models more appropriately reflect the cellular heterogeneity, cell-cell interactions, and molecular divergence. However, the efficiency for PCa organoids and PDXs establishing is relatively low[9, 12]. PCa spheroids based on suitable cell lines are very useful in drug screening as well. Almost all the evaluation indices of drug screening are based on 2D-cultured cells, which showed limitations in 3D drug testing experiments. In this study, we evaluated the susceptibilities of LNCaP cells and spheroids exposed to the same anti-cancer drug.

One of the challenges of spheroids drug testing experiments is to conveniently mass-produce uniformly sized spheroids since the susceptibility for similar-sized spheroids shows convergence[17]. Therefore, we first explored the suitable culture conditions for LNCaP spheroids before evaluating if the formation and size of embedded cultured LNCaP spheroids were consistent. Contrary to some published literature[19-21], our results indicate that 3D-embedded cultured LNCaP cells cannot survive in medium with an FBS concentration lower than 7.5%. The fact that the LNCaP cells were embedded and cultured in Matrigel Matrix, which might act as a barrier between the cells and the whole growth medium, could be the reason for this.

The embedded cultured LNCaP spheroids started to form between the fourth to seventh days and grew over time. Unlike the spheroids formation consistency in round-bottom microplates[17], we found that the embedded cultured LNCaP spheroids in flat-bottom plates had a significant formation inconsistency since the differences in the size of the LNCaP spheroids gradually widened over time.

These results indicate that even though the cells were from the same cell line with the same genotype, the capability of spheroids formation based on single LNCaP cells differed significantly. Compared to d_max_ and lg volume, the spheroids’ volume seemed unsuitable for describing LNCaP cells/spheroids’ size. It is because the large-sized spheroids were not normally distributed, and the small-sized cells/spheroids could not be significantly displayed in the spheroids’ growth curves.

In 3D drug testing experiments, two kinds of 96 well microplates (TC-treated and U-bottom spheroids microplates) are commonly employed. They provide embedded cultured spheroids and floating spheroids, with different drug diffusivity, conditions for cell proliferation, and tightness of the packed cells.

In this study, two drug testing protocols were evaluated for the susceptibility of the 3D-cultured LNCaP cells and spheroids. The first protocol was based on the embedded cultured single LNCaP cells with the same initial plating numbers. According to the spheroids images and distribution results described above, the size of the LNCaP spheroids was not consistent. The spheroids’ size was also restricted by the length of cell cultivation in Matrigel Matrix since Matrigel Matrix becomes unstable after a certain time of culturing.

The other protocol was based on the floating spheroids from U-bottom spheroids microplates. One of the biggest advantages of this method for us was the ability to harvest consistent LNCaP spheroids[17]. Only one floating spheroid could be harvested from each well, and the progress of spheroids formation based on single cells could not be easily observed in this type of microplate.

According to the images of the spheroids exposed to 64 nM docetaxel, the floating spheroids were more sensitive to docetaxel than similar-sized embedded cultured LNCaP spheroids. The LNCaP spheroids formation in the spheroids microplates was based on the cell clusters within a shorter period, dynamic of cell-cell and cell-matrix interaction, all of which still need further evaluation by further experiments.

This part of the study had several limitations. Firstly, we were unable to fully distinguish whether the differences we observed were due to Matrigel Matrix or due to variations in cell-cell and cell-matrix interactions, since the floating spheroids were not embedded in Matrigel Matrix. Secondly, the drug diffusivity, frequency of cell proliferation, and cell maturation times of different kinds of spheroids were not detected in this study, which should be furtherly examined in following projects. Another limitation was that the maximum inhibition of the floating spheroids differed significantly for same-sized spheroids. More experiments with the in the spheroids microplates are needed to confirm our results.

This study described the IC_50_ curves of different sized LNCaP cells/spheroids exposed to docetaxel. The susceptibility of the same cell line to docetaxel should be similar, but we discovered that the IC_50_ values and other parameters differed significantly in different sized LNCaP cells/spheroids. Several studies[22-24] showed that 3D-cultured skin cancer cells showed greater resistance to many types of cytotoxic drugs than 2D-cultured cells in general.

Cancer cell spheroids (CCS) display complete resistance to paclitaxel while demonstrating a nice dose-dependent response in 2D-cultured cells[25]. More interestingly, another study[26] in breast cancer showed that spheroids based on BT-549, Bt-474, and T-47D cell lines were more resistant to paclitaxel and doxorubicin than 2D-cultured cells, while the spheroids based on MCF-7, HCC-1954, and MDA-MB-231 cell lines showed similar drug sensitivities in the corresponding 2D-culture cells. Research[27] revealed that the IC_50_ curves for both 2D and 3D cultures of fast proliferating cells mostly overlap. However, for the slowly proliferating cells, the IC_50_ curves for the 3D cultures attain higher half-inhibitory values. LNCaP cell line is one of the slower proliferating cell lines. Further studies are needed to clarify if those differences in susceptibility could also be detected in fast proliferating PCa cells. Another limitation of these experiments is that the differences in the susceptibilities of different-sized spheroids were evaluated using inconsistently sized spheroids rather than similar-sized spheroids, which also calls for more research.

As previously stated, the same cell line’s susceptibility to the same anti-cancer drug should be similar over a drug concentration course, although different sized LNCaP cells/spheroids may yield different results. So, how should we assess the susceptibility of 3D cultured LNCaP cells/spheroids in order to reflect the actual situation the best?

2D cell culture was introduced as a tool for anti-cancer drug screening in the 1950s[28] and has become an essential part of preclinical drug discovery. 2D-cultured cells are grown as a monolayer in plates and flasks, which provides a flat “full-on-display” structure, different from cells *in vivo*. The drug testing experiments on this cells’ monolayer show higher sensitivity than 3D-cultured cells and PDX models. That is one reason why novel anti-cancer drugs selected by preclinical models have such a low success rate in clinical trials[29, 30].

3D-cultured cells and PDX models provide more *in vivo*-like preclinical models that better mirror *in vivo* responses[31], but the efficiency of PCa organoids or PDX establishment has been relatively low[12, 32]. Spheroids established from PCa cell lines with the same gene characteristics as PCa tissues are another effective preclinical model for anti-cancer drug screening, while appropriate experimental procedures and evaluation indexes are still being developed.

Drug-dose-response curves, which are based on drug testing experiments in 2D monolayer cultured cells, are widely used tools to measure the sensitivity of cells to anti-cancer agents[27]. Similar-sized cancer cells are usually cultured in medium containing uniform drug concentrations. This is done for a long enough period for the cancer cells to be passaged 1-2 times, usually resulting in plunges (around IC_50_ values) in the curves[33, 34]. IC_50_ values were shown to be an imperfect evaluation index in 3D drug testing experiments, implying that a multiparametric evaluation system for spheroids and organoids should be established.

Since there were no standard experimental protocols for assessing spheroids’ susceptibility to anti-cancer drugs, we performed different experiments based on embedded spheroids and floating spheroids. We found that the IC_50_ values of the larger-sized LNCaP spheroids were significantly higher than those of 2D LNCaP cells, 3D-embedded cultured LNCaP cells, and small-sized spheroids. The max inhibition concentration of the drug for spheroids increased with the size of the spheroids, and cell viability did not decrease below 50% when the size of the spheroids was large enough. The R^2^ values of the larger-sized spheroids did not fit the IC_50_ curves.

Because the nonuniformity of the size of spheroids could result in different biological activities, great effort has been made recently in order to produce consistently sized spheroids/organoids in standard labware[17]. However, it is essential to note that, even when we were able to produce similar-sized spheroids, the susceptibility to anti-cancer drug sensitivity could not be evaluated by unfit curves. The maximum inhibition and area under the dose-response curve have been shown to be efficacy parameters for evaluating the susceptibility to drug testing in spheroids[17]. According to our findings, the IC_50_ curves of 3D-embedded cultured LNCaP single cells fit IC_50_ curves, but for the large-sized LNCaP spheroids with a low maximum inhibition and a low R^2^ value, the IC_50_ curves were not suitable to evaluate the susceptibility to the drug.

## CONCLUSION

Our results show that IC_50_ curves in 2D and 3D preclinical models have varied fitness levels. Specifically, IC_50_ curves appear suitable for assessing the sensitivity of 3D single LNCaP cells exposed to docetaxel. However, for large LNCaP spheroids, IC_50_ curves may not be ideal for evaluating the sensitivity of drug test results. This provides a research direction for future drug experiments on prostate cancer and the employment of additional 3D models to simulate the in vivo environment of tumor patients in order to achieve individualized clinical treatment and precision medicine. At the same time, more evaluation indicators (such as maximum inhibition) and experiments (such as spheroid formation) should be investigated and conducted. In addition, the susceptibility of 3D models should be evaluated systematically using several indicators.
