# Corrigendum: IC50: an unsuitable measure for large-sized prostate cancer spheroids in drug sensitivity evaluation

**DOI:** 10.17305/bb.2022.8739

**Published:** 2023-05-01



Corrigendum to: IC50: an unsuitable measure for large-sized prostate cancer spheroids in drug sensitivity evaluation [1].

This corrigendum corrects the authorship information to: Yipeng Xu, Gabriela Pachnikova, He Wang, Yaoyao Wu, Dorothea Przybilla, Zihao Chen, Shaoxing Zhu, Ulrich Keilholz. At the request of Dr. Reinhold Schäfer and with the agreement of all authors, Dr. Reinhold Schäfer is excluded from the list of authors. Dr. Schäfer expresses no doubts about the article's results and conclusion but was erroneously included on the list of authors.

The authors sincerely apologize for the error and confirm that this correction does not change the conclusion of the article.
